# Health and medical experience of migrant workers: qualitative meta-synthesis

**DOI:** 10.1186/s13690-024-01254-z

**Published:** 2024-03-01

**Authors:** Hyun-Jin Cho, Kyoungrim Kang, Kyo-Yeon Park

**Affiliations:** 1 College of Nursing, Pusan National University, Yangsan, Republic of Korea; 2 College of Nursing, Research Institute of Nursing Science, Pusan National University, Yangsan, Republic of Korea

**Keywords:** Migrant workers, Health service, Healthcare, Qualitative research

## Abstract

**Background:**

Migrant workers in Korea are contributing to economic development by resolving labour shortages due to the increase in the ageing population, and they have become necessary and important in the Korean economy. However, long working hours, poor working conditions, and cultural differences can cause migrant workers to experience disadvantages in using health and medical services. Therefore, this study aimed to understand and analyse the health and medical service experience of migrant workers in Korea by reviewing previous studies in order to improve their health and access to medical services.

**Method:**

The qualitative meta-synthesis method suggested by Thomas and Harden was used. The key question for searching the literature is ‘What is the status of the use of health and medical service by migrant workers in Korea and the attributes that affect them?' Five electronic databases (RISS, KCI, KISS, Science ON, and KMbase) were searched for Korean literature published in academic journals until 6 November 2022 using a combination of “migrant worker or foreign worker or foreign labourer or migrant” and “medical or health” in Korean.

**Results:**

A total of nine studies out of 1,006 were included in the review after methodological quality assessment using the Critical Appraisal Skills Program (CASP). The results of studies were synthesised into three themes and ten sub-themes: ‘Personal factors’ of personal health beliefs and the financial burden of healthcare costs; ‘Cultural factors’ of cultural differences in the lifestyle, cultural differences in the healthcare environment, and traditional medicine in the country; and ‘Socio-institutional factors’ of poor and difficult working environment, insufficient information about medical institutions, policies with a lack of practical applicability, systems of healthcare institutions, and healthcare services usage.

**Conclusions:**

This study identified the experiences of migrant workers in using health and medical care services. The results of this study can be used as a basis for improving the health of migrant workers and access to healthcare services. Based on the results of this study, it is necessary to improve the health management ability of migrant workers by developing a health management platform that can support Korean medical information and provide professional and accurate self-health management information.

**Supplementary Information:**

The online version contains supplementary material available at 10.1186/s13690-024-01254-z.

**Table:** 

Text box 1. Contributions to the literature
• Migrant workers in Korea have barriers to using healthcare services due to personal, cultural, and socio-institutional factors, which may negatively impact their personal health.
• This study can provide strategies to improve access to healthcare services for migrant workers in Korea and will contribute to improving their capacities to manage their health.
• This study offers unique insights into the healthcare and medical services for migrant workers in Korea through an integrated review of exploratory studies related to the health and healthcare experiences of migrant workers in Korea.

## Background

The number of foreigners staying in Korea has been on a steady rise despite a brief decline due to COVID-19. Since 2022, the increasing trend has recovered again, with about 2.35 million foreigners staying in Korea as of April 2023, of which about 470,000 were employed either regularly or temporarily [1]. Migrant workers are mainly engaged in labour-intensive jobs such as agriculture, manufacturing, construction, and domestic care, which benefit both countries of origin and migrant countries and contribute to economic growth [2]. Migrant workers have become essential manpower in Korean agriculture and industrial fields while solving labour shortages in the low-wage 3D industry in Korea’s labour market, which is currently ageing, highly educated, and high-wage [3, 4].

However, migrant workers have health problems or health risks due to changes in the living environment and cultural differences [5], and long working hours and poor working conditions cause disadvantages in using health and medical services [4]. According to the “Foreign Workers Survey” conducted by the Korea Institute for Health and Social Affairs in 2020 [6], 72% of the participants said they had been sick over the past year, and 17.9% did not receive hospital treatment even when they were unbearably sick or injured. In addition, the majority of migrant workers reported that their health conditions deteriorated after migration [7]. As the influx of migrant workers in Korea continues to increase, the demand for medical support for migrant workers is also increasing, but access to healthcare is not easy due to social, administrative, and cultural factors [8].

The World Health Organisation (WHO) charter stated that “enjoying the highest level of health that can be achieved without distinction of race, religion, political beliefs, economic or social conditions is everyone’s basic right” [9]. As migration becomes more common, the right to health of migrant workers is a basic right that applies to everyone [10], and more social attention is required for a healthy life and healthy labour [11]. In addition, as Korean health promotion policies are strengthened [12], it is necessary to pay attention to prevention and promotion for the health of migrant workers, and to increase access to practical medical services [11].

To keep up with this trend, there were researched policy-related studies on the health of migrant workers [10, 13, 14], the actual condition of using health information of migrant workers [15], and studies related to the health status and medical service use of migrant workers [16–18]. In this way, efforts are being made to understand the health status, healthcare status, and the status of medical service use of migrant workers in detail and to understand the meaning of participants’ experiences in depth through exploratory research on migrant workers’ health and medical experiences and perceptions [4, 19, 20].

The study attempts to comprehensively understand the status of health management and medical service use experienced by migrant workers through qualitative meta-synthesis [21] based on previous studies that explored the health and medical experiences of migrant workers. In addition, it aims to seek ways to improve the health of migrant workers in Korea and access to health and medical services by deriving and reinterpreting common core concepts from individual studies through synthesis.

## Purpose

The purpose of this study is to comprehensively examine qualitative research related to the healthcare and healthcare service experience of migrant workers in Korea. By understanding and analysing the health management status and healthcare service experiences of migrant workers in Korea, meaningful topics can be synthesised and interpreted, and it is intended to provide fundamental data to improve the health of migrant workers in Korea and access to healthcare services.

## Methods

### Study design

This study is a qualitative meta-synthesis study conducted to synthesise research on the healthcare and healthcare service experiences of migrant workers in Korea.

### Key questions

The key questions for searching the literature are “What is the status of health and medical service use of migrant workers in Korea? " and “What are the attributes that affect their health and medical service use?”

### Literature search, collection and selection process

#### Literature search

This study reviewed only academic papers among Korean literature that studied the healthcare and healthcare service experiences of migrant workers. Authors used five electronic databases to search for all literature corresponding to related subject words without restrictions on the year of publication as a study published in academic journals until November 6, 2022. For the literature search, five Korean databases were searched: Research Information Sharing Service (RISS), Korea Citation Index Quotation Index (KCI), Korean Studies Information Service System (KISS), Science ON, and Korean Medical Paper Database (KMbase). These databases are the most used databases in Korea. We searched for only studies written in Korean to gain a deeper understanding of the meaning of the participants’ statements in qualitative research. To increase the sensitivity of literature search, Google Scholar was searched academic publications to comprehensively include. In addition, additional literature was searched by reviewing the reference lists of the studies obtained through the database search. The main keywords in the databases were searched in Korean. The search terms were the study subjects “migrant worker” OR “foreign worker” OR “foreign labourer”, and to minimise papers that may be omitted from the subjects, “migrant” was additionally searched according to the librarian’s advice, and the study subject “medical” OR “health.” Our detailed search strategy is provided in Additional file 1.

#### Inclusion criteria and limitations

The inclusion criteria were (1) studies on migrant workers in Korea, (2) studies published in academic journals, (3) studies on health and medical experiences, and (4) adult migrant workers. Whereas the exclusion criteria were (1) quantitative studies, (2) conference presentations, abstracts only, dissertations and reports, (3) studies published in languages other than Korean.

#### Literature collection and selection

Studies were collected using electronic databases, and the collected literature was managed using EndNote X9.3.1 (compatible with EndNote 20), a bibliographical management program. The literature selection for the review was performed according to the reporting guidelines recommended by PRISMA 2020 Statement. The literature selection process was carried out by two researchers (HJC, KYP) independently.

**Fig. 1:**
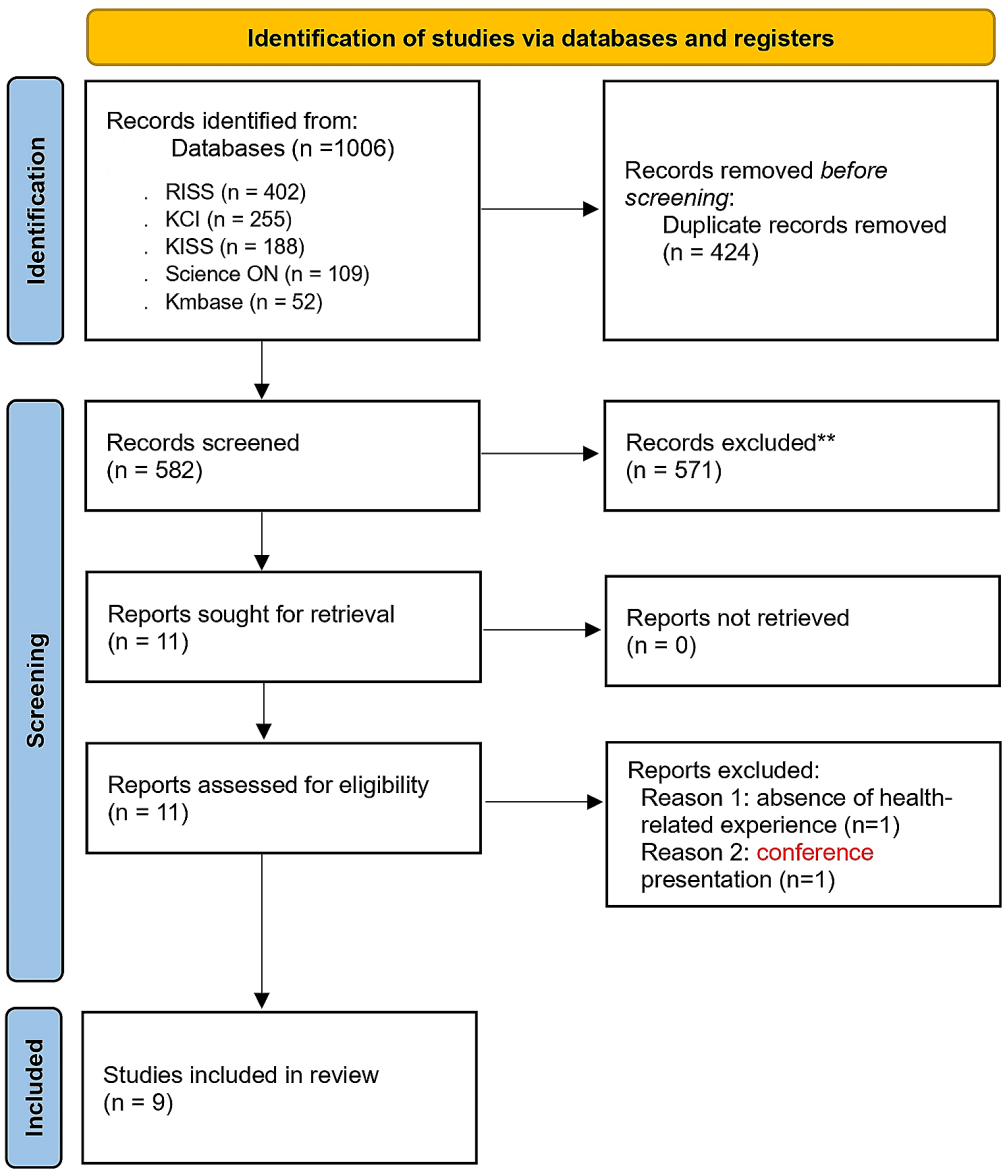
The study selection process using PRISMA 2020

The literature search yielded 1,006 studies, including 402 from RISS, 255 from KCI, 188 from KISS, 109 from Science On, and 52 from KMbase. Using EndNote, 424 duplicate papers were identified and removed. For the remaining 582 articles, titles and abstracts were reviewed according to the inclusion and exclusion criteria, and 571 studies that did not fit the study purpose were excluded. After an in-depth review of the full texts of the selected 11 studies, this study excluded one study that did not specify the health-related experience of the study participants, and one conference presentation. Consequently, nine studies were selected for the systematic review and have been identified in full text. Three researchers (KK, HJC, KYP) independently performed the literature selection process to ensure the validity and reliability of the results. During this process, researchers addressed disagreements by reviewing the manuscript through a research meeting and adjusting until an agreement was reached. The literature excluded in the selection stages was recorded and the document selection process was described using the 2020 PRISMA systematic review flow chart (Fig. 1).

### Quality assessment

The quality evaluation of the literature was performed using Critical Appraisal Skills Program (CASP) tool [22]. The CASP qualitative research checklist is commonly used to assess qualitative research in meta-synthesis studies [23, 24]. This checklist is a tool for evaluating the reliability, integrity, and rigour of the literature. The CASP checklist consists of ten questions in three sections: ‘Are the results of the study valid?‘, ‘What are the results?‘, and ‘Will the results help locally?‘. The quality appraisal tool includes clear statements of aims, appropriateness of qualitative methodology, appropriateness of research design, appropriateness of recruitment strategy, data collection, the relationship between researcher and participants, ethical issues, rigour in data analysis, clear statement of findings, and value of the research. Most questions require ‘yes/no or can’t tell’ response. The higher the score on the CASP, the more systematic the qualitative research is conducted, scores were presented as the percentage of questions [25]. Two researchers (HJC, KYP) independently evaluated a quality of the literature, and if there was a disagreement during the quality evaluation process of the literature, three researchers (KK, HJC, KYP) resolved disagreements through discussions and consensus. The CASP evaluation results of nine studies were more than 80% (Table 1).

### Data extraction

By analysing the characteristics of the literature included in this study, data were extracted by authors, publication years, study purpose, characteristics of participants, study methodology, data collection methods, and major results (Table 1). Data extraction was carried out independently by two researchers (HJC, KYP), and if opinions disagreed during the data extraction process, three researchers (KK, HJC, KYP) reached an agreement through discussion.

### Data synthesis

The thematic synthesis method of Thomas and Harden [21] was used to synthesise the data of the selected literature, and it was analysed in three steps: the coding of text ‘line-by-line’, the development of ‘descriptive themes’, and the generation of ‘analytical themes’ [21]. This approach was chosen as it could address key questions, with the aim of providing information to improve the health of migrant workers. It has also been used commonly in qualitative meta-synthesis studies [26–30].

- The first step: It extracted and coded meaningful sentences and phrases while repeatedly reading statements or study results of study participants in nine articles.
- The second step: While discussing the similarities and differences in the coded data, similar codes were categorised into descriptive sub-themes.
- The third step: Analytic themes were created to provide clarified meaning around the list of sub-themes.
- If there was a disagreement between researchers, it was resolved through discussion in the process of analysis and synthesis.

**Table 1:** Descriptive Summary of Included Studies

Author Year	Aim	Participant Characteristics	Data collection/ Methodology	Results	CASP
Lee et al. 2013 [31]	Exploring situations that might put the middle-aged Korean-Chinese female migrant workers at risk for work-related musculoskeletal diseases (WMSDs)	23 Middle-aged Korean-Chinese female migrant workers	Focus groups and semi-structured interview/ Directed content analysis	1. Risk factors in work-related musculoskeletal diseases 1) Physical risk factors 2) Socio-psychological factors: Discrimination and distrust, lack of autonomy in work, employment insecurity 2. Major health problems and healthcare access limitations among middle-aged Korean-Chinese female migrant workers	100
Lim et al. 2014 [32]	Examining the meanings and perceptions of ‘clinic centre’ given by both heath care givers like medical specialists and students and heath care receivers like migrant workers	Total 28 15 migrants (Including migrant workers), five medical specialists from the centre, two centre steering committee members, two college nursing volunteers, and four high school student volunteers.	Participant observation and in-depth interview/ Ethnography	1. Constructing care providers’ understanding and meaning of ‘clinic centre’ 2. Understanding ‘clinic centre’ from a migrant perspective: Migrants perceive ‘clinic centre’ as spaces where they are only interested in health promotion activities, regardless of their immigration status	80
Li 2014 [33]	Exploring the reality of physical pain in the migrant labour process and the specific process of becoming invisible and the structural aspects of them “voluntary” overuse of the body.	Eight Middle-aged Korean-Chinese Women Migrant workers	Participant observation and focus group interview/ Life history analysis	1. The physical pain of migrant labour 2. Invisible realities of body pain 1) Body pain situated in life: gendered labour and body experiences in Chinese society 2) Uncovered workers’ compensation and exclusion from the health insurance system 3) Personalised treatment and the sociocultural construction of illness 4) ‘Spontaneous’ body abuse and the delay of body pain	90
Kim 2015 [4]	Examining the conception and factors that affect the utilisation of healthcare services among foreign migrant workers in Korea	Nine migrant workers	Focus group interview/ Qualitative content analysis - deductively (Andersen-Newman Behavioural Model)	1. Predisposing factors: Demographic factors, social structure, health beliefs 2. Enabling factors: Individual/household level (means and methods of access to health care), community level 3. Need factors: Perceived need, evaluated need	100
Kim et al. 2016 [5]	Examining what medical experience international marriage migrant women have and what cultural differences and conflicts they face in their new society, Korea.	Total 11 Nine international marriage migrant women (workers) Two administrative workers at the public health centre in Ansan	Focus group interview and the minutes of official meetings/ Narrative analysis	1. Korean healthcare system for migrants 2. Married migrant women’s experiences of Korean healthcare and cultural conflicts: Communication problems and solutions, lack of understanding of detailed departments of medical system, difficulties in accessing general hospitals and cultural differences, dissatisfaction with doctor-patient interaction, cultural differences in prescription and perception of drugs, cultural differences in emergency care.	90
Shin et al. a 2019 [20]	Examining the health management process of undocumented migrants and identifying potential barriers preventing them from using health and medical care	14 undocumented migrants (Including migrant workers)	In-depth interviews/Grounded theory	1. Causal condition: Economic factors, labour environment factors, information accessibility factors 2. Contextual condition: Difficulties experienced by being undocumented 3. Phenomenon: Poor health, low healthcare utilisation 4. Intervening condition: Language availability, interaction with healthcare providers 5. Action/Interaction: Service satisfaction 6. Consequence: Healthcare utilisation prospects	90
Shin et al. b 2019 [34]	Understanding the health status of undocumented migrants and find ways to improve their access to health and medical services	12 experts who have provided medical assistance for migrants for more than five years and the literature review	In-depth interviews and the literature review/ methodological triangulation and researcher triangulation	1. Insufficient medical services 2. Communication problems 3. Lack of information 4. Need for establishing a separate health care system 5. Need for substantial and systematic free medical centres 6. Importance of utilising the community 7. Situation where available health and medical services are concentrated in Seoul 8. Improving the environment surrounding undocumented migrants 9. Improving cultural discrimination (Muslim issues)	100
Chun 2021 [19]	Exploring the health management experience of Vietnamese married immigrant women living in the city	11 Vietnamese immigrant women residing in the urban area (Including migrant workers)	In-depth individual interviews/ Grounded theory	1. Core category: Health is not a necessity but a choice in a strange land called Korea 2. Contextual condition: The hard thing—exposing “myself” to the world, medical services hard to access even in a state of illness 3. Causal condition: Unfamiliar life to live alone 4. Action-interaction: Health pushed away in turbulent life 5. Intervening condition: Power to prioritize health 6. Consequence: Health in the chain with life	100
Son et al. 2022 [35]	Examining healthcare service delivery and immigrant health behaviours	17 stakeholders from the public and non-governmental institutions and organisations for immigrant healthcare services	In-depth interview/Thematic analysis	1. Contraction of healthcare delivery and use 1) Worsening access to healthcare due to reduced mobility and discrimination 2) Quarantine policies and contraction of healthcare supply 2. Weakened medical care continuity 1) Delays in disease treatment and management 2) Reduced medical support and limited communication	100

## Results

### Characteristics of the included literature

A total of nine studies [4, 5, 19, 20, 31–35] were included in this review. The included literature was published between 2013 and 2022, and the total number of study participants recruited were 133. The methods of data collection were two focus group interviews [4, 31], three in-depth interviews [19, 20, 35], one participant observation and in-depth interviews [32], one participant observation and focus group interviews [33], one literature review and in-depth interviews [34], and one meeting minutes and focus group interviews [5]. In addition, the methods of data analysis were two grounded theories [19, 20], one directed content analysis [31], one ethnography [32], one life history analysis [33], one deductive content analysis [4], one narrative analysis [5], one triangulation [34], and one thematic analysis [35].

### Meta-synthesise

As a result of synthesising research on the healthcare and healthcare service experience of migrant workers in Korea, the following three themes were derived: (1) personal factors, (2) cultural factors, (3) social institutional factors (Table 2). Because migrant workers prioritise work before health, they choose work despite poor working conditions, and suffer from physical and mental health problems due to hard work, changes in living environment, and cultural differences, but they bore the pain by force or reduce the pain in their own way after self-diagnosis because of a society that restricts access to medical services. They used healthcare services centred on medical support projects or free clinics, but they did not reveal that they were sick even when they were sick, and repeated the vicious cycle of threatening their health with a work-centred life pattern to show that their body is a productive labour force.

**Table 2:** Themes of Health and Medical Experience of Migrant Workers

Themes	Sub-themes
Personal factors	Personal health beliefs Financial burden of medical expenses
Cultural factors	Cultural differences in living conditions Cultural Differences in Medical Environment Folk remedies in one’s country
Social institutional factors	Poor and hard-working conditions Insufficient information about medical institutions Policies with a lack of practical applicability Medical institution’s system Use of healthcare services

#### Personal factors

##### Personal health beliefs

Migrant workers immigrated to Korea to find jobs for making money. He entered poor working conditions to find a job in Korea, and showed an addiction to investing his body and time with the idea that it would be over if he failed to work due to the currency value several times higher than his home country [33]. Migrant workers should not be sick to take responsibility for the living expenses of their families in their home country, and even if they were sick, it was difficult to pay for medical expenses and receive treatment [32]. Migrant workers overworked their bodies with constant labour to show that their bodies were a productive labour force, revealing physical pain was difficult to reveal diseases because it meant they had to quit work, and continued labour by connecting the cause of the disease to ageing or underlying diseases [33]. In the process of enduring hard labour by dismissing migrant labour in Korea as temporary labour to make a lot of money and return home [33], they were difficult to take care of their health even if there were signs of health problems because they have been pushed out of their priorities in life [19]. Migrant workers delayed taking care of their health by comforting themselves with the fact that they could work despite physical or psychological pain [33], or they hoped that their family or time would solve their health problem because they could not solve it themselves [19]. Some migrant workers have health belief that make them reluctant to use hospital due to distrust of western medicine, which become a threat to their health [4].

##### Financial burden of medical expenses

Migrant workers were financially burdened with medical and insurance costs, and they were unable to actively use medical services, such as giving up treatment when hospital costs such as examination or surgery were high [4, 20, 31, 32]. Especially, unregistered migrant workers gave up the use of medical services due to their low ability to pay for health insurance due to an environment where they had no visas and low wages [20]. If it was possible that support from friendly medical staff and medical expense support groups, healthcare services could be revisited, but few organisations provide full medical expenses, so it was difficult to solve high treatment costs such as large surgery alone [20].

#### Cultural factors

##### Cultural differences in living conditions

Migrant workers were at risk of exposure to various diseases due to stress and long hours of work under a different weather, food, and language from their home countries [19, 32]. Migrant workers consumed a lot of sugar such as cakes, resulting in type 2 diabetes, and their bodies were ruined by junk food and high-calorie food-oriented eating habits [34]. In the case of married migrant women, physical health problems were experienced due to the food culture of their in-laws different from their home countries, and hard work instead of their husbands with physical and economic difficulties in foreign country [19]. In the case of married migrant female workers, it was invisible that postpartum depression, loneliness, and homesickness, so they could not ask for help, and they also developed diseases by enduring health abnormalities due to lack of Korean language skills [19]. In some cases, medical staff forced Muslim women to take off hijab even though they did not interfere with the examination [20], and the conflict felt by migrant workers due to cultural differences did not end up as an inconvenience, but as a health threat [5]. It also had difficulty choosing appropriate medical institutions or using medical services as language barriers regardless of gender [4, 20, 32, 34].

##### Cultural differences in medical environment

The experience of using medical services different from the home country made it reluctant to reuse medical services, and cultural differences in the medical environment acted as a factor that threatened the health of migrant workers [5]. Migrant workers felt psychological burdens and difficulties in the process of using general hospitals in Korea due to their experiences in using medical services different from their home countries. They felt anxious about treatment due to a lack of understanding of the detailed divisions of the Korean medical community and confusion arising from institutional differences, and confidence in hospitals decreased [5]. Unlike in their home country, migrant workers felt frustrated because they did not know the causes and treatment methods of their health problems, as well as the types and effects of medicines due to the unilateral doctor’s treatment method [31], the lack of explanations about medicines at pharmacies, and felt like discriminated against [5]. They also asked for a prescription of drugs or fluids that worked in their home country, but they felt humiliated when their doctor refused or advised them to stop taking the medicine they had taken previously taken [5]. In addition, traditional first aid methods vary from country to country, which also became a conflict factor in the process of interaction with Korean doctors, making them hesitant to reuse medical services [5]. Migrant workers had restrictions on the use of healthcare services due to much greater psychological stress due to limitations of language communication disorders and cultural differences in the use of medical services [5].

##### Folk remedies in one’s country

Migrant workers purchased and took health supplements through their acquaintances or used folk remedies such as moxibustion treatment [20, 33], and used them for emergency treatments experienced in the cultural environment of their home country [5]. When they were sick, they took drugs brought from their home country after self-diagnosis [33], received help from religious institutions or religious leaders, and found a shaman due to unknown symptoms [4]. They also exercised such as stretching, hiking, and cycling [20].

#### Social institutional factors

##### Poor and hard-working conditions

It was difficult for migrant workers to use hospitals on weekdays because they were concerned that employers would negatively view the use of medical institutions in migrant workers mainly working in difficult jobs with poor working conditions [4, 20, 31, 32]. As a result, since most symptoms were tolerated and preventive medical services were not available [4], even if there was a health problem, it had become a factor that threatens the health of migrant workers because they cannot respond properly. Public hospitals and private medical support organisations that can provide medical support are concentrated in Seoul [34], so geographical access to medical services was low. When employed, they experienced psychological burdens and stress due to discrimination caused by being limited to jobs avoided by Koreans, distrust of migrant workers, tension that cannot be free to eat or rest due to walking on eggshells around their boss, and job insecurity [31]. In particular, unregistered migrant workers experienced emotional pain, psychological atrophy, disability caused by industrial accidents, depression and despair, employers’ distrust of physical pain, and inappropriate follow-up measures as illegal residents [33]. Migrant workers were tired of mental stress from long-term labour, and physically felt pain in their limbs, neck, thyroid gland, abdomen, and kidneys [20]. Female migrant workers experienced physical burdens and the risk of musculoskeletal diseases due to the act of hugging children while working as a housekeeper and restaurant employee, repeated handling of heavy tableware, long standing or uncomfortable posture, repeated use of hands and wrists, long working hours, and lack of rest [31, 33].

##### Insufficient information about medical institutions

Migrant workers were not aware of information probably related to the use of medical services such as medical expenses, medical departments, examination status, and use procedures because there was no place to obtain information on medical institutions [4, 20, 31, 34]. Even if there was health insurance, they were not aware of how to use it or benefits, and they were not aware of emergency medical services or free medical services, so there was a limit to the use of health medical services [4]. Migrant workers obtained health information through family, friends, acquaintances, employers, and migrant communities [4, 33], but in the case of communities and communal groups, uncertain information was supplied with commercial medical information, and the necessary information was not well provided [4, 34]. Migrant workers also obtained health information through TV or the Internet [19, 31]. Migrant workers use medical services based on their own description on disease condition or symptoms rather than expert diagnosis or advice, so they often rely on personal information from acquaintances when choosing a medical institution [4].

##### Policies with a lack of practical applicability

- Industrial accident.

In the case of unregistered migrant workers, it was rare to treat for diseases or accidents caused by industrial accidents when using medical institutions, and they rarely received health check-ups or supported medical expenses at work [20]. In the event of an industrial accident, compensation can be applied for, but in the process to deal with industrial accidents, employers were fined for hiring unregistered migrants, and unregistered migrants could be forcibly repatriated for illegal stay, making it almost impossible to deal with industrial accidents [20]. In addition, there was an institutional loophole in which migrant workers could not apply for industrial accidents at all when they first entered the country and start working [34]. In the case of female migrant workers, they usually worked at small business offices or private homes that did not have industrial accident insurance, and their physical pain was not visible because it was a chronic disease that was not included in industrial accident [33].

- Health insurance policy.

Migrant workers were very passive in signing up for health insurance, considering migrant labour as a short-term temporary labour [33], and the requirements for joining medical insurance should be confirmed residency for more than three months, and for migrant workers who are unfamiliar with life in Korea, the differential medical welfare system according to their status of residence felt discriminatory and became a factor threatening their health [5]. In the case of medical insurance, not only unregistered migrant workers, but also migrant workers employed in occupations such as nursing labour, domestic labour, and farms were excluded [33].

- COVID-19 disinfection policy.

Korea’s quarantine policies during the pandemic of infectious diseases such as COVID-19 had a more negative impact on migrants’ use of medical services and were fatal to unregistered migrants [35]. As the medical support project for migrants was suspended, it became difficult to receive medical expenses as well as disease treatment, and the increase in hate discrimination against migrants during the COVID-19 pandemic also reduced access to medical services [35]. The continuity of healthcare deteriorated as hospitals were reluctant or refused to visit migrants, and hospitals were delayed even if they were sick [35]. The quarantine policy due to COVID-19 has blocked the continuity of medical care in the early detection and treatment of migrants and the management of chronic diseases, acting as a factor that threatens the health of migrants [35].

##### Medical institution’s system

Migrant workers had difficulties in using medical services due to the lack of medical institutions that could interpret [4, 19]. In the process of using medical services, if the migrant worker did not communicate well with the medical staff, the medical staff became annoyed, discriminated against, or refused treatment [20]. As a result, emotional accessibility was lowered, which made it difficult to use medical services [4]. Unregistered migrants felt uncomfortable and anxious about filling out documents related to personal information when using medical services and were reluctant to reuse medical services because they had been denied access to public health centres or other medical institutions because they did not have a visa [20].

##### Use of healthcare services

Migrant workers mainly used free clinics for migrants, clinics near workplaces, and health centres [19, 20, 31]. Migrant workers used free clinics for migrants due to economic burden [32], or health centres or cultural centres that provided free examinations and treatment [19]. It was difficult to get medical treatment during weekday working hours, so they visited a health centre on Sunday due to the burden of treatment costs [32] or were treated under someone else’s name to receive medical insurance benefits [19]. Migrant workers want to receive Western treatment, but have used low-cost oriental medicine, or purchased non-prescription drugs at pharmacies [4, 20]. Migrant workers want to receive Western treatment, but have used low-cost oriental medicine, or purchased non-prescription drugs at pharmacies [4, 20]. Most of them also did not use the dentist clinic with high-cost burden due to the large number of non-benefit treatments [20]. In the case of unregistered migrants, they received simple medical treatment at the free clinic of the support centre and, in the worst case, they planned treatment in their home country, where they could benefit from health insurance [20]. Migrant workers who had economic support, were able to communicate in the language, or met friendly medical staff were optimistic about using medical services. On the other hand, migrant workers gave up using medical services, endured pain, and thought about returning to their home countries if they had experiences of not receiving economic support, language communication difficulties, and treatment being rejected by medical staff [20].

## Discussion

This study aimed at enhancing the well-being of migrant workers in Korea and increasing their access to healthcare and medical services by performing qualitative meta-synthesis related to the health and medical experience of migrant workers in Korea. Nine studies [4, 5, 19, 20, 31–35] were included in the final analysis of this reviews in this study, the healthcare and medical service experiences of migrant workers in Korea were examined systematically. Through the integration of these studies, three main themes and ten sub-themes were derived, namely: ‘personal factors,’ ‘cultural factors,’ and ‘social and institutional factors.’

The first theme, ‘Personal factors’, was derived into three sub-themes: ‘individual health beliefs’ and ‘economic burden of medical expenses.’ Migrant workers moved for the purpose of earning money, so they prioritise work over health. According to the study of Lee [33], migrant workers’ postponement of healthcare while overworking themselves in voluntary labour was described as “self-rule based on economic principles.” Even if the purpose of migration is to gain economic benefits through labour, migrant workers need to take a long-term perspective and shift their mindset of health priorities so that their bodies can be maintained as a productive workforce. Therefore, it is necessary to periodically implement preventive programs or education on healthcare for migrant workers so that distrust of Western medicine or false health beliefs can be changed. Migrant workers also were unable to receive medical treatment on time due to economic burdens, and it is necessary to provide various medical services by expanding professional free medical services.

‘Cultural factors’ are factors that face from differences from other environments while leaving the home country, regardless of the country of migration. For example, migrant workers, who had low sugar intake in their home countries, ate a lot of sugar such as cakes that could be easily encountered in Korea, and suffered from chronic diseases such as diabetes [34], and not only they were unfamiliar with Korean food culture, but also they were experienced health problems [34] due to a diet focused on cheap and convenient junk food or high-calorie food [36]. Since this dietary problem is the factor of threat that can be prevented through food and health education, providing rest and health education to migrant workers through game-based health workshops at the corporate level will effectively eliminate this factor and greatly enhance motivation for healthcare. Differences in eating habits as well as different medical service systems from their home countries are cultural factors that confuse migrant workers. It will be possible to increase the accessibility of Korean medical services by providing basic knowledge to migrant workers who are confused by the medical service system that is different from their home country by explaining detailed areas of Korean medical service [5]. Recently, Korea is changing into a multicultural society due to the influx of migrant workers to fill the labour shortage and the increase in international marriage, so it is necessary to improve multicultural acceptance to understand and recognise members of different cultures [37]. In line with the social trend, medical staff need to provide an appropriate explanation of the treatment process of the disease with an understanding of migrant workers [20], and culture nursing education should be provided for medical staff so that nursing in consideration of culture can be conducted [38].

In the context of ‘social and institutional factors,’ the poor working environment serves as a fundamental cause of health problems among migrant workers, and it is difficult to use medical institutions without improvement in the working environment where they can freely use medical institutions even if health and medical services are improved and expanded [20, 34]. As a result, it is important to create a social atmosphere that guarantees the basic rights of migrant workers, and it is also essential to reduce psychological burdens and stress through cooperative relationships among co-workers rather than maintaining the employer-employee relationship. Moreover, migrant workers had limited access to medical services due to lack of information on medical institutions. To solve this difficulty, it is important to utilise a community for information sharing [34], and it is necessary to provide information on medical institutions by guiding institutions and organisations that provide free medical treatment upon arrival. On the other hand, although industrial accident compensation insurance, health insurance systems, and COVID-19 quarantine policies were not applied discriminatively to migrant workers, the social system experienced in a foreign country acted as a factor limiting migrant workers’ use of medical services. These systems also were not consistently applied to migrant workers due to unfamiliar foreign policies, which in turn restricts their access to medical services. Local governments where many migrants live proposed measures to support health and medical services to supplement these policy problems. For example, the study of Shin and Choi [34] were proposed the centres would offer free professional support across various departments tailored to the needs and scale of the migrants, or ‘Health Card’ was created to use a certain amount of insurance in a way that does not expose the identity for undocumented migrant workers within the local government. Migrant workers did not communicate well with medical staff due to language barriers, and as a result, unfriendly medical staff and refusal of treatment had become a factor that threatens the health of migrant workers. To solve this problem, the study of Shin and Choi [34] was suggested that migrants who can use Korean well for smooth communication could be trained as activists or interpreters to help migrants use medical services. Additionally, it is crucial to develop hospital information and educational materials in multiple languages, enabling nurses to provide care and to establish dedicated departments catering to foreigners, thereby streamlining the treatment process for migrant workers [38].

This study examined the health and medical experiences of migrant workers based in the preceding literature. When faced with physical and psychological distress, their initial response often involved enduring the discomfort and waiting for a natural recovery, while seeking health-related information through various ways, including family, acquaintances, communities, and the Internet. These behaviours led to ways such as self-diagnosis based on inaccurate information, taking health supplements or medications, using folk remedies such as moxibustion, using first aid methods of home country, exercising, or seeking out shamans. They try to use medical services after their symptoms or pain worsens, but due to financial burden, they mainly use free clinics, public health centres, and oriental medicine clinics. This pattern of healthcare coping results in the disease getting worse due to missing the appropriate treatment period. To break this pattern of healthcare coping, it needs to be provided that accurate information about disease symptoms. For this, it is important to develop a healthcare program or platform for migrant workers to enable self-health management. It also can improve migrant workers’ ability to cope with health problems that providing professional and accurate information, such as information about Korean medical institutions or disease-related information. Especially, simple and repetitive health information can be provided easily, quickly and accurately at the right time by using an easily accessible platform such as a chatbot.

Studies have been examined to determine the status of migrant workers’ healthcare or use of medical services and their health and medical care experiences, but these results have not been integrated. This study has significance in that it provides comprehensive and integrated data on the health and medical experiences of migrant workers by applying a qualitative meta-synthesis method to study that qualitatively analysed the health and medical experiences of migrant workers in Korea from various perspectives. It is also meaningful in that it provided basic data for the development of health management platforms and policy activation to improve the health of migrant workers in Korea and access to health and medical services. However, this study is limited in that it was unable to analyse in detail from each perspective, even though the study participants had diverse perspectives, including not only migrant workers but also medical staff, volunteers, and public officials. In addition, studies included in the qualitative meta-synthesis were examined from the perspective of female migrant workers, so there are limitations in comparing them with the health and medical experiences of male migrant workers. Accordingly, follow-up qualitative studies on the health and medical experiences of male migrant workers will be needed. This study may have a publication bias by reviewing only studies written in Korean to understand in depth the meaning of the statements made by the participants in individual studies. However, we tried to reduce publication bias by performing a quality assessment of the literature, searching for various synonyms in Korean approved databases, and adding grey literature and manual searches. Nevertheless, this study only reviewed studies of migrant workers in Korea to integrate the health and healthcare service experiences, so it may not be applicable to other countries with different socio-cultural backgrounds. It is suggested that future studies integrate the health and healthcare service experiences of migrant workers in other countries and compare them with Korea.

## Conclusion

This study conducted qualitative meta-synthesis based on exploratory studies related to the health and medical experiences of migrant workers in Korea to comprehensively understand the healthcare and healthcare service experience of migrant workers in Korea. The final nine studies were integrated, as a result, personal factors such as individual health beliefs and economic burdens on medical expenses, cultural factors such as cultural differences between living and medical environments, private therapy in the home country, and social and institutional factors such as poor and hard-working conditions, insufficient information about medical institutions, policies with a lack of practical applicability, the system of medical institutions, and social and medical service use. Through this, it was confirmed that the health management status of migrant workers and their experience in using medical services. The results of this study could be used as basic data for improving the health of migrant workers in Korea and improving accessibility to healthcare services in the future. Based on the results of this study, it is suggested to develop a health management platform that can provide Korean medical information to migrant workers and provide professional and accurate self-health management information to improve their health management skills and increase the use of healthcare services.

### Electronic supplementary material

Below is the link to the electronic supplementary material.

Supplementary Material 1

## Data Availability

The datasets generated during and/or analysed during the current study are available from the corresponding author upon reasonable request.

## Abbreviations

WHO: World Health Organization
RISS: Research Information Sharing Service
KCI: Korea Citation Index Quotation Index
KISS: Korean Studies Information Service System
KMbase: Korean Medical database
PRISMA: Preferred Reporting Items for Systematic Reviews and Meta-Analysis
CASP: Critical Appraisal Skills Program
