# Supplementary material for: Health and medical experience of migrant workers: qualitative meta-synthesis
Source: Arch Public Health. 2024 Mar 1;82:27. doi: 10.1186/s13690-024-01254-z (PMC10905938; doi:10.1186/s13690-024-01254-z)
Supplement: Supplementary file 1 — Supplementary Material 1 [file 13690_2024_1254_MOESM1_ESM.docx]

**Search Terms**

(The English in brackets is for understanding purposes and the search is in Korean only.)

Search terms: "이주 노동자 OR 외국인 노동자 OR 외국인 근로자 OR 이주민" AND "의료 OR 건강"

( "migrant worker OR foreign worker OR foreign labourer OR migrant " AND "medical OR health" )

DATABASE: KMbase, KCI, KISS

Date Searched: 06.11.2022.

Strategy:

1. 이주 노동자 (migrant worker)

2. 외국인 노동자 (foreign worker)

3. 외국인 근로자 (foreign labourer)

4. 이주민 (migrant)

5. 1 OR 2 OR 3 OR 4

6. 의료 (medical)

7. 건강 (health)

8. 6 OR 7

9. 5 AND 8

DATABASE: RISS, Science ON

Date Searched: 06.11.2022.

Strategy:

1. 이주노동자 | 외국인노동자 | 외국인근로자 | 이주민

(migrant worker | foreign worker | foreign labourer | migrant)

2. 의료 | 건강 (medical | health)

3. 1 AND 2
